# Refractive errors and risk factors for myopia in infants aged 1–18 months in Tianjin, China

**DOI:** 10.1186/s12886-021-02172-2

**Published:** 2021-11-23

**Authors:** Lu Huo, Yuanyuan Qi, Shaozhen Zhao

**Affiliations:** 1 Tianjin Key Laboratory of Retinal Functions and Diseases, Tianjin Branch of National Clinical Research Center for Ocular Disease, Eye Institute and School of Optometry, Tianjin Medical University Eye Hospital, Tianjin, China; 2 Tianjin Women’s and Children’s Health Center, Tianjin, China

**Keywords:** Refractive error, Myopia-related risk factors, Infants

## Abstract

**Background:**

Infancy is the of a child’s visual development. Refractive errors, especially myopia, are a common vision disorder. Thus, the purpose of this study was to explore refractive errors and risk factors for myopia among infants aged 1–18 months in Tianjin, China.

**Methods:**

A total of 583 infants aged 1–18 months participated in this cross-sectional study at Tianjin Women’s and Children’s Health Center in China from February 2019 to November 2020. Each infant received a complete ophthalmologic examination, and myopia-related risk factors were investigated using a questionnaire.

**Results:**

A total of 583 eligible infants participated in this study, including 312 (53.5%) boys and 271 (46.5%) girls. There were 164 (28.1%) premature born infants. The mean age was 6.59 ± 4.84 months (range, 1–18 months). The mean spherical equivalent (MSE) for the right eye was 1.81 D ± 1.56 D, with no difference related to sex (*P* = 0.104). Refractive state showed an average hyperopia of +2.74 ± 1.74 D at early ages, followed by a trend toward less hyperopia, finally reaching +1.35 ± 1.44 D at the age of 18 months (P ≤0.001). The overall prevalence rates of myopia (MSE ≤ −0.50 D), emmetropia (−0.50 D<MSE<+0.50 D), hyperopia (MSE ≥ +2.00 D), and astigmatism (≥ 1.50 D) were 5.1%, 10.8%, 42.7%, and 49.9%, respectively. The chi-square tests showed that gender, gestational age ≥37 weeks, winter birth, prenatal exposure to environmental tobacco smoke, and parental history of high myopia were associated with children’s myopia (*P* = 0.022, *P* = 0.023, *P* = 0.038, *P* = 0.015, P<0.001, respectively).

**Conclusions:**

Among Chinese infants in Tianjin, hyperopia and astigmatism were the most frequent refractive errors, and the diopter was lower in individuals with higher age. In a small number of infants with myopia, genetic factors and the prenatal environment were associated with the early onset of myopia.

## Background

Previous studies [1, 2] have shown that the refractive state in neonates is hyperopic, and different powers in different meridians of the cornea result in relatively high astigmatism. The physical characteristics of ocular structure are largely based on the optical components losing refractive power as the eye enlarges and compensating for the enlargement, a process known as emmetropization [3]. In this way, hyperopia and astigmatism gradually decrease with age, thereby leading to the ideal refractive state for humans. Animal studies have demonstrated that visual experience has an important role in determining refractive development and that visual deprivation predictably produces large refractive errors in the process of emmetropization [4, 5]. Refractive errors are among the most common causes of visual impairment in children. They have been associated in a severity-dependent manner with strabismus and amblyopia [6]. For early intervention, it is necessary to understand the starting point and the corresponding refractive error at the different stages of development [7].

Although hyperopia or astigmatism remains a major risk factor for amblyopia in different ethnicities and geographic regions [8], myopia boom is growing globally, and 49.8% of the world population is predicted to be myopic in 2050 [9]. Although the genetic contributions to the growth of the eye are indisputable, there are also polygenic interactions where the phenotypic expression relies heavily on environmental conditions. During the past decade, many epidemiologists have studied the risk factors for myopia. Rupal et al. have found that from 3 years of age and onward, greater time spent outdoors is associated with a reduced risk of incident myopia in the Avon Longitudinal Study of Parents and Children (ALSPAC) [10]. The education intensity [11], near-work activities [12, 13], electronic devices [14, 15], ethnicity, and parental socioeconomic status [13, 16, 17] are also positively associated with myopia in school-age children.

Most clinical research on infancy myopia that has focused on premature babies grouped them by the degree of retinopathy of prematurity (ROP), which is referred to as myopia of prematurity (MOP). The severity of ROP at different stages or occurrence of retinal scars has been shown to increase the risk of developing significant MOP [18–20], whereas the corrected gestational age (CGA) is negatively associated with high myopia in school-age children and even adults compared with full-term children. Recent studies have indicated that early-onset myopia is becoming more common in full-term infants. For example, a study in Hong Kong has shown that the prevalence of 3- to 6-year-old children myopia increased from 2.30% in 1996–1997 to 6.32% in 2006–2007 [21]. The prevalence of myopia has been reported to be 6.6% among African-American and 3.7% among Hispanic children in Los Angeles County [22], and 3% among children aged 1–48 months in Boston [23]. Most importantly, the earlier the onset of myopia, the more likely high myopia will develop [24]; this trend alone supports the need for vision screening among preschool children every year [25]. Infants do not have the burden of schoolwork, use of electronic devices, or near-work activities. It is still unknown whether there is a combination of genetic traits or environmental factors that contribute to the occurrence of myopia early in life, similar with teenagers.

This study had two main purposes: (1) to determine refractive errors in infants and toddlers; (2) to analyze the risk factors for myopia in this population through the investigation of biological, nutritional, and environmental factors in early life.

## Methods

This was a cross-sectional study. A total of 583 children were recruited on a voluntary basis at the Tianjin Women’s and Children’s Health Center from February 2019 to November 2020. All of them came to the hospital to undergo congenital cataract examination. A three-stage, stratified, cluster sampling method was used for the selection of participants. First, two districts were randomly selected from the six main urban districts in Tianjin. Second, two primary communities were randomly chosen from each district. Finally, 650 children were selected continuously during the recruitment period, and 583 children participated (89.6% response rate). Children with previous congenital cataract, glaucoma, eye tumor, strabismus, or ptosis, and those children who did not cooperate were excluded. Additionally, premature infants with ROP were excluded from this study. Moreover, the infants recruited in this study had not been treated by intravitreal injection or other cryotherapy, so as to represent the relationship more accurately between myopia and preterm birth itself, rather than ROP.

Before cycloplegic refraction, an anterior chamber depth shadow test was performed to ascertain that pupil-dilating drops were safe to use for the child. Cycloplegic refraction was induced with three drops of tropicamide (1%) instilled 10 minutes apart. Cycloplegia was then evaluated after an additional 20 minutes. If a light reflex was still detected, another drop of tropicamide was administered. Refractive error was measured using a Spot Vision Screener (SVS, Welch Allyn Spot, VS100). In a dimly lit room, the image acquisition by the Spot was done at a distance of approximately 1 meter while the infant was in a sitting position or when the infant’s head was placed on the parent's shoulder. The device was kept at the infant’s eye level, and red or green light displays and sound were used to attract the infant’s attention. All the procedures were performed by the same optometrist. The average of the three measurements was taken as the final result.

### Questionnaire

Both parents were asked to complete a questionnaire to collect children’s basic information, including name, gender, date of birth, gestational age (GA) as determined from the date of last menstrual period, birth weight (BW), type of delivery (normal vaginal birth/cesarean birth), and whether the child was a twin.

Preterm birth was defined as birth before 37 weeks of gestational age. Low birth weight was defined as birth weight < 2500 g.

Potential myopia-related factors were selected based on a thorough literature review. The following were the main data collected: (1) Tianjin is a city in northern China with four distinct seasons, so months of birth were also grouped to four seasons: spring (March–May), summer (June–August), autumn (September–November), and winter (December–February); (2) nightlight in the bedroom (yes/no); (3) the type of infant feeding in the frist 6 months of age (breastfeeding, breastfeeding and formula milk, formula milk); (4) smoking exposure, defined as at least one cigarette per day for 1 year or longer; Chinese families live mainly in groups, and family members include parents and four grandparents; thus, passive smoking history was positive if at least one of the family members smoked during pregnancy (yes/no); (5) maternal reproductive age (<25 years, 25–35 years, >35 years); (6) parental history of myopia.

Refractive error was recorded as the mean spherical equivalent (MSE), which was defined as the spherical power plus half of the negative cylinder in the diopter (D) unit. Myopia was defined as MSE ≤ −0.50 D, emmetropia as −0.50 D<MSE<+0.50 D, and hyperopia as MSE ≥ +2.00 D. Astigmatism was defined as a cylindrical measurement (≥ 1.50 D) and the classifications were based on minus cylinder notation. The distribution of orientation of the astigmatism was stratified into three categories: With-the-Rule Astigmatism (WTR, cylinder axes between 0° and 30°, or between 150° and 180°), Against-the-Rule-Astigmatism (ATR, cylinder axes between 60° and 120°), and Oblique Astigmatism (cylinder axes between 30° and 60°, or between 120° and 150°).

### Statistical analysis

Statistical analysis was performed using SPSS for Windows, version 26.0 (IBM-SPSS, USA). The Pearson correlation coefficient of MSE from the right eye and the left eye was 0.863. Only the right eye data were presented. The components of refraction were analyzed for significant variation with age. Comparisons of the prevalence rates of refractive errors were performed using the chi-square test. Multivariate logistical regression analysis of the association between independent risk factors and myopia was conducted, and odds ratios (ORs) and 95% confidence intervals (95% CIs) were calculated. All statistical tests were two-sided, and statistical significance was defined as *P* < 0.05.

## Results

### General Information

In this study, a total of 583 infants were finally enrolled. There were 312 boys (53.5%) and 271 girls (46.5%), and the mean age of these infants was 6.59 ± 4.84 months (age range, 1–18 months). The GA at birth was 37.38 (±2.61) weeks, and BW was 2950.52 (±709.02) g. Among the participants, there were 164 premature infants (28.1%) and 419 full-term infants (71.9%).

### Distribution of mean refraction error by age

Table 1 shows that the subjects were divided into five groups according to age since birth. The MSE of all the children was 1.81 D (±1.56, 95% CI 1.68–1.94). The MSE of the right eye of boys and girls was 1.91 D (±1.60, 95% CI 1.73–2.10) and 1.69 D (±1.52, 95% CI 1.52–1.87), respectively. The MSE of boys was more hyperopic than that of girls during every period (*P*=0.104).

**Table 1:** Distribution of MSE in the right eye according to age and gender

Age in months	N	Mean	SD	95% CI for mean	
				Lower bound	Upper bound
1–3	140	2.74	1.74	2.45–3.03	
3–6	143	2.02	1.39	1.79–2.25	
6–9	156	1.26	1.28	1.05–1.46	
9–12	73	1.27	1.23	0.95–1.53	
12–18	71	1.35	1.44	1.01–1.69	
Total	583	1.81	1.56	1.68–1.94	
*P*	<0.001				
Boys					
	312	1.91	1.60	1.73–2.10	
1–3	77	2.87	1.93	2.45–3.30	
3–6	78	2.10	1.29	1.79–2.38	
6–9	76	1.30	1.19	1.02–1.59	
9–12	46	1.37	1.33	1.03–1.77	
12–18	35	1.42	1.51	0.89–1.97	
Girls					
	271	1.69	1.52	1.52–1.87	
1–3	63	2.58	1.50	2.19–2.93	
3–6	65	1.93	1.51	1.58–2.29	
6–9	80	1.22	1.37	0.94–1.54	
9–12	27	1.04	1.04	0.63–1.45	
12–18	36	1.28	1.38	0.84–1.75	
*P*	0.104				

Table 2 provides the overall prevalence of myopia, emmetropia, hyperopia, and astigmatism. Among the 583 right eyes, stratified by age and sex, astigmatism was the most prevalent (49.9%), followed by hyperopia (42.7%), and emmetropia (10.8%), while the infants with myopia accounted for 5.1%.

**Table 2:** Prevalence of refractive errors in 1–18-month-old children determined by Spot Vision Screener

Parameters	Myopia	Emmetropia	Hyperopia	Astigmatism
Age in months	n (%)	n (%)	n (%)	n (%)
1–3	4 (2.9%)	7 (5.0%)	102 (72.9%)	66 (46.4%)
3–6	4 (2.8%)	13 (9.1%)	77 (53.8%)	86 (60.1%)
6–9	12 (7.7%)	22 (14.1%)	40 (25.6%)	89 (57.7%)
9–12	4 (4.1%)	12 (16.4%)	14 (19.2%)	25 (34.2%)
12–18	6 (8.5%)	9 (12.7%)	16 (22.5%)	25 (35.2%)
Total	30 (5.1%)	63 (10.8%)	249 (42.7%)	291 (49.9%)
*P*	0.190	0.045	<0.001	<0.001
Boys	10 (3.2%)	37 (11.9%)	140 (44.9%)	146 (46.8%)
Girls	20 (7.0%)	26 (9.6%)	109 (40.2%)	145 (53.5%)
*P*	0.035	0.380	0.258	0.106

Hyperopia (≥+2.00 D) was present in 72.9% of the infants between 1 and 3 months, and there was a trend to lower prevalence of hyperopia in older individuals than in younger infants (P<0.001); hyperopia in boys was present in 44.9%, compared with 40.2% in girls, but sex differences were not observed (*P* = 0.258).

As the most common refractive error, the overall prevalence of astigmatism (≥ 1.50 D) was 49.9%. Similar to hyperopia, the prevalence of astigmatism was lower in older infants (P< 0.001); the prevalence of astigmatism in children aged 1–3 months was 46.4%, which was higher than that in children aged 12–18 months (35.2%; *P* < 0.001). In children aged 1–18 months, WTR astigmatism, ATR astigmatism, and oblique astigmatism accounted for 72.1%, 24.1%, and 3.8% of all the orientations of astigmatism, respectively. The proportions of individuals with the three orientations of astigmatism were lower in older individuals (Table 3).

**Table 3:** Distribution of orientations of astigmatism in children aged 1–18 months

Parameters	With-the-rule astigmatism	Against-the-rule astigmatism	Oblique astigmatism
Age in months	n (%)	n (%)	n (%)
1–3	48 (35.0%)	14 (10.1%)	4 (2.9%)
3–6	66 (46.2%)	17 (11.9%)	3 (2.1%)
6–9	62 (39.7%)	24 (16.0%)	3 (1.9%)
9–12	15 (20.5%)	9 (12.3%)	1 (1.4%)
12–18	19 (26.8%)	6 (8.5%)	0 (0%)
Total	210 (72.1%)	70 (24.1%)	11 (3.8%)
*P*	0.002	0.453	0.696

The prevalence of myopia was 7.7% in infants with 6–9 months of age , then lower in those with 9–12 months (4.1%), and again high in children aged 12–18 months (8.5%). When we defined myopia as at least −1.00 D, the prevalence lower to 2.7% (Table 4). There was a significant difference in the prevalence of myopia (*P* = 0.035) or MER of at least −1.00 D (*P* = 0.020) between boys and girls.

**Table 4:** Prevalence of myopia by different criteria

Parameter	Myopia categories	
	(SE at least −0.50 D)	(SE at least −1.00 D)
Age in months	n (%)	n (%)
*P*	0.129	0.793
1–3	4 (2.9%)	4 (2.9%)
3–6	4 (2.8%)	2 (1.4%)
6–9	12 (7.7%)	6 (3.8%)
9–12	4 (4.1%)	2 (2.7%)
12–18	6 (8.5%)	2 (2.8%)
Total	30 (5.1%)	16 (2.7%)
Gender		
*P*	0.035	0.029
Boys	10 (3.2%)	4 (1.3%)
Girls	20 (7.0%)	12 (4.4%)

### Risk factors associated with myopia

In univariate analysis (Table 5), the prevalence of myopia was significantly associated with gender (*P* = 0.022), GA ≥ 37 weeks (*P* = 0.023), winter birth (*P* = 0.038), family members’ smoking history during maternal pregnancy (*P* = 0.015), and parental high myopia (P<0.001). According to the multivariate logistical regression analysis (Table 6), the risk of myopia was higher in winter-born than in summer-born children (adjusted OR 1.31, 95% CI 0.53–3.08, *P* = 0.017); another risk factor was the family members’ smoking history during maternal pregnancy (adjusted OR 2.47, 95% CI 1.10–5.53, *P* = 0.027). The presence of high myopia among parents was another risk factor for developing myopia (adjusted OR 4.27, 95% CI 1.15–15.80, *P* = 0.029). Furthermore, compared with the reference group (children with no myopic parents), the OR for developing myopia was 4.71 (95% CI 0.69–31.86, *P* = 0.112) and 6.78 (95% CI 1.51–30.34, *P* = 0.012) for children with one or two myopic parents, respectively.

**Table 5:** Characteristics of the study participants and myopia prevalence (Univariate)

Parameters	Total,n(%)	Myopia,n(%)	No Myopia,n(%)	*P*
Total	583 (100%)	30 (5.1%)	553 (94.9%)	
Gender				0.022
Boys	312 (52.2%)	10 (3.2%)	302 (96.8%)	
Girls	271 (47.8%)	20 (7.4%)	251 (92.6%)	
GA				0.023
≥37weeks	419 (71.8%)	27 (6.4%)	392 (93.6%)	
<37weeks	164 (28.2%)	3 (1.8%)	161 (98.2%)	
BW				0.808
≥2500g	456 (78.2%)	24 (5.3%)	432 (94.7%)	
<2500g	127 (21.8%)	6 (4.7%)	121 (95.3%)	
Type of delivery				0.912
Normal vaginal birth	247 (42.3%)	13 (5.3%)	234 (94.7%)	
Cesarean birth	336 (57.7%)	17 (5.1%)	319 (94.9%)	
Twins				0.114
Yes	74 (12.7%)	1 (1.4%)	73 (98.6%)	
No	509 (87.3%)	29 (5.7%)	480 (94.3%)	
Birth Season				0.038
Spring	101 (17.3%)	6 (5.9%)	95 (94.1%)	
Summer	135 (23.1%)	1 (7.1%)	134 (99.3%)	
Autumn	155 (16.7%)	8 (5.2%)	147 (94.8%)	
Winter	192 (32.9%)	15 (7.8%)	177 (92.2%)	
Ambient Nighttime Light Exposure				0.719
Yes	232 (39.7%)	11 (4.7%)	221 (95.3%)	
No	351 (60.3%)	19 (5.4%)	332 (94.6%)	
Infant feeding				0.859
Breastfeeding	237 (40.6%)	13 (5.5%)	224 (94.5%)	
Breastfeeding mixed Formula milk	226 (38.9%)	12 (5.3%)	214 (94.7%)	
Formula milk	120 (20.5%)	5 (4.2%)	115 (95.8%)	
Smoking during pregnancy: any parents				0.015
Yes	192 (32.9%)	16 (8.3%)	176 (91.7%)	
No	391 (67.1%)	14 (3.6%)	377 (96.4%)	
Maternal age at birth				0.537
<25y	12 (2.1%)	0 (0%)	12 (100.0%)	
25-35y	444 (76.1)	25 (5.6%)	419 (94.4%)	
>35y	127 (21.8%)	5 (3.9%)	122 (96.1%)	
Parental high myopia				<0.001
Yes	114 (19.6%)	16 (14.0%)	98 (86.0%)	
No	469 (80.4%)	14 (3.0%)	455 (97.0%)	
Number of myopic Parents				0.257
None	103 (17.6%)	4 (3.9%)	99 (96.1%)	
One	246 (42.1%)	17 (6.9%)	229 (93.1%)	
Two	234 (40.3%)	9 (3.8%)	225 (96.2%)

**Table 6:** Results of the Multivariate Logistical Regression analysis of the prevalence of myopia and systemic parameters

Parameters	Odds Radio	95% CI	*P*
Gender			
Boys	1 [Reference]		
Girls	0.67	0.20-2.26	0.526
GA			
≥37weeks	1 [Reference]		
<37weeks	0.51	0.08-2.96	0.457
Birth Season			
Spring	0.66	0.36-1.92	0.828
Summer	1 [Reference]		
Autumn	0.19	0.05-0.63	0.418
Winter	1.31	0.52-3.08	0.017
Smoking during pregnancy: any parent			
No	1 [Reference]		
Yes	2.47	1.10-5.53	0.027
Parental high myopia			
No	1 [Reference]		
Yes	4.27	1.15-15.80	0.029
Number of myopic Parents			
None	1 [Reference]		
One	4.71	0.69-31.86	0.112
Two	6.78	1.51-30.34	0.012

### Discussion

In our study, the predominant refractive state of infants from 1 to 18 months in each age group was hyperopia. As expected, hyperopia was lower in older children, reflecting the eye growth. This finding is similar to the results of previous studies [26–28]. A recent, large, longitudinal study in Berkeley [27] has shown that, during the first 2 years of life, there is a developmental process of the eyeball that matches the eye’s optical power to its axial length so that the unaccommodated eye is focused at distance. Emmetropization is caused by a combination of multiple mechanisms [29]. After the age of 1 or 2 years, neonatal hyperopia or myopic refractive error rapidly decrease and turn into emmetropia [30]. Morgan [31] suggested that, unlike in animal models, where an accurate 0 degree is the aim, emmetropia is not a developmental end point in children; rather, it is mild hyperopia. Our data showed that the average refractive error was mild hyperopia (1.35 ± 1.44 D) at 18 months, and the process of emmetropization can be considered a convergence of refractions toward a low hyperopic value.

As shown in Table 2, hyperopia (≥ +2.00 D) was observed in 72.9% of the newborns, stratified by age and sex. Among 1–18-month-old children, the overall prevalence of hyperopia of the right eye was 42.7%. By the same standards, the prevalence was higher than that observed in Hispanic whites (35.1%, 6–12 months) [22] and Lumpur (33.3%, 6–11.9 months) [24], and lower than that among Singaporean Chinese [32] children (7.5%, 6–11.9 months), suggesting that Singaporean children could have higher risk of myopia in the future. Although it has been well-established that hyperopia decrease with infants’ age, Ingram and coauthors [33] found the prevalence of hyperopia (> +2.0 D) to be 11.0% in the first years of life and even 12.2% at the age of 3 years. Atkinson [34] suggested a substantial variability in the extent of emmetropization within the hyperopic group. In his longitudinal study, the initial MSE of 8-month-old children was +1.9±0.8 D in the control group and +4.6±0.5 D in the hyperopic group; the final MSEs of the control and hyperopic groups were +1.4 D and +3.0 D at 36 months, respectively. It is possible that accommodation reduction in high hyperopia affects the emmetropization process, so that a certain percentage of children do not emmetropize and thus remain hyperopic.

It may be that the prevalence of astigmatism has a wide range of values due to different criteria, sample populations, and ethnic groups, with the prevalence ranging from 3.8% to 70% [35] in preschool children. In the present study, we focused exclusively on refractive astigmatism and did not discuss internal (or lenticular) astigmatism. As for astigmatism with the highest rate of refractive error, the overall prevalence of astigmatism (≥−1.50 D) in our study was 49.9%, and that in infants aged 3–6 months was 60.1%, which was higher than that in infants aged 12–18 months (35.2%). Generally, corneal astigmatism is due to the anterior and posterior surfaces and occurs when the shape of the cornea is toroidal [36]. Isenberg [37] has reported an average of six diopters of corneal astigmatism in newborn babies. As infants grow older, an emmetropization of the astigmatic refractive error occurs, the corneal curvature gradually flattens, and astigmatism tends to be stable [38]; however, astigmatism remains and even increases with age in individual cases. Fan et al. [39] reported that astigmatism of 1.00 D or more was found in 29.4% of children aged 3 years, and ≥31% in 6 year-old children (*P* = 0.470). These results suggest that astigmatic blur might influence emmetropization and may even signify a failure of normal emmetropization. This means that parents must pay close attention to children’s vision at the age of 1.5 years to avoid the potential risk of amblyopia and need for special follow-up. According to Gwiazda, most children have ART astigmatism before the age of 4 years; after that age, as eyelid pressure increases, the existing ART gradually changes to WRT astigmatism [40]. However, in our study, WTR astigmatism was prevalent during infancy, and this type of astigmatism showed the greatest differences between the age groups (35.0%, 1–3 months; 26.8%, 12–18 months); yet, its proportion was still the highest among all three astigmatism categories at 18 months. There was no evidence that the oblique astigmatism differed between age groups, and ART was the most stable, which is consistent with the report by Mitchell et al [41]. Our findings might suggest that ART is more persistent and WTR more likely to resolve.

The association between astigmatism and refractive errors is controversial. Some studies [42] have found a positive correlation between astigmatism and hyperopia in preschool children. In contrast, some prospective studies have demonstrated that astigmatism can induce myopia development and progression in older children [43, 44]. However, higher astigmatism suggests a combined impact of genetic predisposition, smoking [45], screen

exposure [46], and video games [47]. More longitudinal and large-scale studies are needed to explore the correlation between astigmatism and the progression of refractive errors.

In our study, the overall prevalence of myopia (SE ≤ −0.50 D) in the right eye was 5.1%, with two peaks evident in the 6–9-month-old group and 12–18-month-old group at approximately 7.7% and 8.5%, respectively. This was a relatively low prevalence compared with that reported in the study of Singaporean Chinese children [32], where the prevalence of myopia was 15.8%, 14.9%, and 20.2% in children aged 6–11.9, 12–23.9, and 24–35.9 months, respectively. Our findings (≥ −1.0 D, 2.7%) were similar to those reported for Hispanic children (3%), higher than those for non-Hispanic White children (1%), and lower than those for African-American children (6%) at the age of 6–72 months [48]. In another prospective, multiethnic, cross-sectional study [24], myopia was not found in infants aged 6–11.9 months and 12–17.9 months. Myopia (≥ −1.00 D) was found in 1.3% of the population in 24–36-month-old Kuala Lumpur, Malaysia children (*n* = 151). Our report indicated that the rate of occurrence of myopia (≥ −1.00 D) among Chinese infants aged 1–18 months was similar to that reported in the North America. However, in school-age children, the prevalence and development rate of myopia in Asian children are higher than those in European countries. The education level [49, 50] and the exposure to screens [51] have been determined as independent risk factors for myopia.

In our research, the prevalence of myopia fluctuated among the age groups, but without reaching statistical significance (P=0.190). A study of Singaporean Chinese children [47] has shown an unusual pattern, in which the prevalence of myopia was 15.8%, 8.6%, and 6.4% in children aged 6–11.9, 36–47.9, and 60–72 months, respectively. One of the reasons could be that the process of emmetropization may take place as early as during the first few years of life. Another reason may be that table-mounted autorefractor was used as the primary method of measuring refractive error in children aged 24 months or older; in contrast, 6–24-month-old children were assessed using Retinomax autorefractor (Nikon), which overestimated the myopic component of refraction in young children. A similar situation was reported in American children [48], where a long decreasing trend from 12 months of age was found; such observation cannot be attributed simply to the use of a lower concentration of cyclopentolate in children younger than 12 months and may actually reflect active emmetropization.

We intended to determine factors in early life that induce myopia. We identified a winter birth, prenatal exposure to environmental tobacco smoke, parents having high myopia, and two myopic parents as factors with increased odds of myopia in infants aged 1–18 months.

The birth season has also been linked to myopia, based on the relationship between the duration and intensity of light exposure and myopia. Natural light exposure stimulates the retina to secrete more dopamine, which is a key to controlling eye growth and preventing myopia [52]. Tianjin is located in the northern hemisphere. Our report revealed that 15 of 30 infants (7.8%) born in winter were myopic, which was 15-fold higher compared with those born in summer (*P* = 0.038). Myopia was significantly associated with being born in winter (adjusted OR 1.31, 95% CI 0.52–3.08, *P* = 0.017). This suggests that the lack of daylight exposure in winter may lead to myopia; in other words, children spend more time outdoors during summer than in winter. However, a cross-sectional study (5653) including 1222 children aged 0–3 years in eastern China showed that children born in winter had a reduced risk of developing myopia (OR 20.11, 95% CI 20.25–20.03, *P* = 0.01). These results should be considered with caution because Ma et al. [53] used non-cycloplegic refraction to measure refractive error on account of overestimating myopia due to uncontrolled accommodation in infants. In addition, the risk factors for myopia in school-age children are more complex, and Donovan [54] has provided additional evidence that temporal variations in the progression of myopia among school-age children occur during the course of a school year. but this still needs to be validated by more experiments.

Despite extensive and long-term studies, the relationship between myopia and parental smoking varies from study to study. The pathological basis of the former research was the neurotransmitter acetylcholine, which acts through either nicotinic acetylcholine receptors (nAChRs) or muscarinic receptors to target the tissues of the eye and regulate the refractive development [55]. Nicotine activates nAChRs and is a constituent in tobacco smoke, and the impact of passive smoking on refractive error development may vary at various stages of life [56]. In the Singaporean children (STARS) study [57], a population-based study of Chinese children aged 6–72 months, maternal history of ever smoking, maternal history of smoking during the child’s life, and paternal history of smoking showed lower ORs of childhood myopia (OR 0.50, 95% CI 0.30–0.84, *P* = 0.01; OR 0.39, 95% CI 0.20–0.76, *P* = 0.01; and OR 0.72, 95% CI 0.54–0.96, *P* = 0.02, respectively). Several studies have evaluated only parents as sources of passive smoking in the family; however, our questionnaires included all the members who came into close contact with the infant, including the grandparents. Our results showed that smoking during pregnancy have more risk of myopia and lower hyperopic after controlling for all confounding variables (adjusted OR 2.47, 95% CI 1.10–5.53, *P* = 0.027) compared with infants whose parents never smoked. Similarly, in a prospective birth cohort study among 572 children (mean age 3 years) in Asia [58], it was shown that those whose family members had smoked from birth to before 6 months had a greater myopia prevalence compared with nonexposed children (adjusted OR 2.79, 95% CI 1.24–6.29, p=0.01), but no association with mean SE or AL was found in children exposed to passive smoking. Due to the complex pharmacological effects of nicotinic acetylcholine receptors, This might explain the conflicting results of different studies.

Children whose parents had high myopia were analyzed by univariate or multivariate logistical analysis; both analyses revealed a significant positive association between the prevalence of high myopia in parents and the child's myopia (adjusted OR 4.27, 95% CI 1.15–15.80, *P* = 0.029). Also, children with one or two parents having myopia had a higher risk of myopia when compared with those whose parents did not have myopia (OR 4.71, 95% CI 0.69–31.86, *P* = 0.112; OR 6.78, 95% CI 1.51–30.34, *P* = 0.012, respectively). Numerous previous studies, including prospective cohort [59], cross-sectional [60], case-control studies [61], and meta-analysis [62], support these results. Previous studies [60, 63] have demonstrated that myopic parents could share the same environment and lifestyle; for example, parental high education, especially in mothers, can lead to less outdoor activities and more reading time for children. This also suggests that parents need to provide children with a healthy environment and lifestyle for growth. In the present study, children were too young to have near-work so the genetic factors could be better reflected if the above confounding factors were excluded.

Although preterm birth (GA ≥37 weeks) was associated with myopia in univariate analysis (P=0.023), it was not statistically significant in multivariate logistical analysis (P=0.457) in our study. Varghese [64] reported that GA was not associated with myopia but with BW. In the present study, children with LBW (BW < 2500 g) were almost twice as likely to have myopia as those with BW ≥ 2500 g (OR 1.87, *P* = 0.441), but crucial evidence is still lacking. We suspect that the reason may be that gestational age at birth only has an early effect on the diopter values of preterm infants, and the growth and development of the premature have reached the full-term state when we recruited them. Fielder [65] proposed a hypothesis that myopia was probably the normal refractive state in infants before full-term, but in this study, we missed that stage. Moreover, only three of 164 premature infants had myopia (1.82%, 3/164), among the 30 myopic infants in our study. In the future, more participants are needed to discuss the refractive status of premature infants at different postnatal ages in a longitudinal study.

Cycloplegic retinoscopy is widely used to measure refractive errors in children. However, retinoscopy is time-consuming, examiner-dependent, and associated with a steep learning curve. In 2013, the AAPOS published guidelines for screening of amblyopia risk factors (AARFs) [66]. The SVS has been used in many studies because it is more convenient, flexible, portable, and does not require more active cooperation. De Jesus [67] evaluated the accuracy of Spot Vision Screening among 134 patients aged 17–50 years and reported that the difference between SVS and subjective clinical refractometry under cycloplegia expressed in SE was +0.66±0.56 D. De Jesus considered the difference nonrelevant in a clinical setting, supporting the use of SVS as an ancillary method for estimating refraction. Qian et al. [68] found a strong linear relationship in SE between Spot and retinoscopy (Pearson's r=0.95, P<0.01) in Chinese children aged 4–6 years. In addition, the Spot provided good specificity and sensitivity in detecting amblyopia risk factors according to 2013 AAPOS criteria [69], and it is very common in communities in China.

Potential limitations of our study should be noted. First, we used tropicamide (1%) to reach a healthy level of cycloplegia before Spot Vision Screener. Although cyclopentolate (0.5%) and atropine (0.5%) are better cycloplegic agents than tropicamide (1%), they also produce systemic and local adverse events due to their muscarinic antagonist activity; for example, there is a higher risk of gastric atony. Hence, we chose to use tropicamide in our study for safety purposes, but we did not observe any residual accommodation in this study. Second, myopia-related factors were reported by parents in this questionnaire. Although this method has widely been used in previous studies, the deviation of parents’ memory may lead to recall bias. Third, this was a cross-sectional study, and the association between myopia-related factors and myopic refraction does not necessarily indicate a true cause–effect relationship. Finally, our sample only represents the level of Tianjin urban area, and there was a volunteer effect. our conclusions should be confirmed in a longitudinal study in the future.

## Conclusions

In summary, in 1–18-month-old Chinese infants included in this study, the most common refractive errors were hyperopia (≥ +2.00 D) and astigmatism (≥ −1.50 D); both showed a significantly lower prevalence in older infants. This study’s subjects were ideal because they had not been exposed to any educational pressure during the early ages, which provides evidence for a winter birth, prenatal exposure to environmental tobacco smoke, parental high myopia history, and biparental myopia as independent risk factors for myopia of infants. Unlike in older children, genetic factors and prenatal environment, and not necessarily the postnatal environment, are associated with early-onset myopia.

## Data Availability

De-identified data are available upon request to the first author.

## Abbreviations

MOP: Myopia of prematurity
CGA: Corrected gestational age
ROP: Retinopathy of prematurity
GA: Gestational age
BW: Birth weight
MSE: Mean spherical equivalent
WTR: With-the-rule
ATR: Against-the-rule
CI: Confidence interval
D: Diopter
SD: Standard deviation
OR: Odds ratio
95% CI: 95% confidence intervals
